# Near-surface processing on AlGaN/GaN heterostructures: a nanoscale electrical and structural characterization

**DOI:** 10.1186/1556-276X-6-132

**Published:** 2011-02-11

**Authors:** Giuseppe Greco, Filippo Giannazzo, Alessia Frazzetto, Vito Raineri, Fabrizio Roccaforte

**Affiliations:** 1 Consiglio Nazionale delle Ricerche - Istituto per la Microelettronica e Microsistemi (CNR-IMM), Strada VIII n. 5, Zona Industriale, 95121 Catania, Italy; 2 Scuola Superiore di Catania, University of Catania, Piazza dell'Università, 2, 95124, Catania, Italy

## Abstract

The effects of near-surface processing on the properties of AlGaN/GaN heterostructures were studied, combining conventional electrical characterization on high-electron mobility transistors (HEMTs), with advanced characterization techniques with nanometer scale resolution, i.e., transmission electron microscopy, atomic force microscopy (AFM) and conductive atomic force microscopy (C-AFM). In particular, a CHF_3_-based plasma process in the gate region resulted in a shift of the threshold voltage in HEMT devices towards less negative values. Two-dimensional current maps acquired by C-AFM on the sample surface allowed us to monitor the local electrical modifications induced by the plasma fluorine incorporated in the material.

The results are compared with a recently introduced gate control processing: the local rapid thermal oxidation process of the AlGaN layer. By this process, a controlled thin oxide layer on surface of AlGaN can be reliably introduced while the resistance of the layer below increase locally.

## Introduction

Gallium nitride (GaN)-based heterostructures are promising materials for the fabrication of high-frequency and high-power devices. In particular, the presence of spontaneous and piezoelectric polarization charges in AlGaN/GaN layers leads to the appearance of a two dimensional electron gas (2DEG) at the AlGaN/GaN interface, typically having sheet carrier densities *n*_s _approximately 1 × 10^13 ^cm^-2 ^and high mobility (1,000-1,500 cm^2^/V s) [1]. These properties make the materials suitable for the fabrication of transistors based on the 2DEG operating at high frequencies (up to tens of gigahertz), i.e., high-electron mobility transistors (HEMTs).

**Figure 1:**
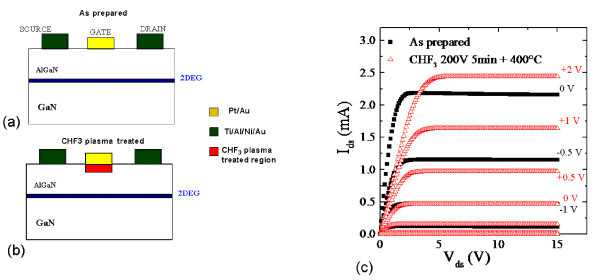
**Schematic representations**. Schematic representations of an untreated HEMT device **(a) **and of a HEMT subjected to CHF_3 _plasma processing **(b)**. *I*_DS_-*V*_DS _characteristics of HEMT device not subjected to the plasma treatment (squares) and subjected to the plasma treatment and to an annealing (triangles).

In Figure 1a, a schematic of a typical HEMT device is reported, in which the location of the 2DEG at the interface between GaN and the AlGaN barrier layer is reported. The current flow between the source and drain Ohmic contacts is controlled modulating the 2DEG carrier concentration in the channel region through the bias applied to the gate Schottky contact on the AlGaN barrier layer.

To date, for many applications, conventional AlGaN/GaN HEMTs have been fabricated as "depletion mode" transistors, i.e., these have a negative threshold voltage (*V*_th_) [2]. However, the next generation of devices will require a more efficient use of the electric power. Hence, enhanced mode (normally-off) AlGaN/GaN HEMTs have become more desirable because these offer simplified circuitry (eliminating the negative power supply), in combination with favourable operating conditions for device safety.

Achieving reliable normally-off operation in AlGaN/GaN HEMTs is a challenging goal of current GaN technology. Several solutions, mostly involving nanoscale local modifications of the AlGaN barrier layer (e.g., recessed gate process [3], fluorine-based plasma etch [4], surface oxidation [5], etc.) have been recently proposed. Clearly, the transport properties of the 2DEG at AlGaN/GaN interfaces are strongly affected by those processes. In this context, using advanced nanoscale-resolution characterization methods can be the optimal way to monitor these local changes and to fully assess the basic transport phenomena in AlGaN/GaN heterostructures, in order to ultimately achieve reliable devices.

The accurate control of the threshold voltage (*V*_th_) is a key issue for normally-off HEMTs fabrication. In fact, several physical parameters affect the value of the threshold voltage *V*_th _[6], like the Schottky metal/semiconductor barrier height (*Φ*_*B*_), the thickness of the AlGaN barrier layer (*d*), the residual doping concentration in the AlGaN (*N*_*D*_), the polarization charge at the AlGaN/GaN interface (*σ*) or the concentration of charges intentionally introduced in the AlGaN barrier (*N*_*F*_).

The introduction of negative charges in the near-surface region of the AlGaN barrier can be a possible method to monitor the carrier sheet concentration of the 2DEG and, hence, the value of *V*_th_. Based on this idea, *Cai et al. *[4] demonstrated the possibility to shift the threshold voltage of AlGaN/GaN HEMTs to positive values by introducing fluorine ions by means of a reactive ion etching plasma process in CF_4._. However, this process introduces a large amount of defects in the AlGaN barrier layer, which can lead to a degradation of the 2DEG mobility. Hence, an annealing process, after the gate fabrication, is needed to repair the damage and recover the mobility. The use of other plasma techniques, like inductive coupled plasma (ICP), could be also considered to reduce the damage and better control the parameters defining the normally-off operation (threshold voltage and sheet carrier concentration of the 2DEG).

A reduction of the barrier thickness *d *leads also to a positive shift of *V*_th_, as reported in the conventional approach of the recessed gate [2]. Typically, recessed gate structures are formed by selective plasma etchings [7]. However, etching just a few nanometers can be extremely difficult particularly considering a high reproducibility and wafer uniformity. Alternatively, Chang et al. [8] reported, in the case of AlN/GaN heterostructures, that a near surface oxidation process can be useful to convert into Aluminum oxide a surface-layer of AlN and, then, to reduce the thickness of the barrier layer below the critical thickness.

Other experiments investigated the effects of a thin oxide layer on the surface of AlGaN using a plasma treatment in O_2 _or in N_2_O [5]. In this context, the effects of a rapid thermal oxidation on the surface were not addressed yet.

In this context, this work studies the effects of near-surface processing on the properties of AlGaN/GaN heterostructures, combining conventional electrical analyses of HEMTs with advanced nanoscale characterization techniques as transmission electron microscopy (TEM), atomic force microscopy (AFM) and conductive atomic force microscopy (C-AFM). In particular, nanoscale current measurements demonstrated a local reduction of the leakage currents (i.e., an increasing of the resistance of the material) both using a CHF_3 _plasma or rapid oxidation treatments of the surface. Hence, these processes could find interesting applications in the fabrication of innovative GaN-based transistors.

### Experimental

AlGaN/GaN heterostructures grown on different substrates (SiC, Si, Al_2_O_3_) were used in our experiments. In order to determine the physical properties of the 2DEG, HEMTs devices with an appropriate geometry were fabricated. First, reference HEMT devices (i.e., not subjected to the plasma treatment) were fabricated. Source and drain Ohmic contacts were formed by an annealed Ti/Al/Ni/Au multilayer [9] and the gate Schottky contact was subsequently formed by a Pt/Au bilayer [9]. To study the effect of the plasma treatment on the 2DEG transport properties, the region where the gate electrode had to be fabricated was modified (before metal deposition) with a plasma process using a CHF_3_/Ar gas mixture, as schematically illustrated in Figure 1b. The plasma treatment was performed at room temperature using the Roth & Rau Microsys 400 ICP equipment. The CHF_3_/Ar gas flux was 20 sscm and the operating pressure in the chamber was 5 × 10^-2 ^mbar. The control bias, the power, and the process duration were 200 V, 250 W and 300 s, respectively. Afterwards, the Pt/Au gate electrode was formed on the same region subjected to plasma treatment, using a self-aligned process and lift-off technique for metal definition. Finally, the sample was subjected to an annealing process at 400°C, in order to recover the damage induced by the plasma process. It is worth noting that this annealing process does not cause degradation of the gate Schottky contact.

In order to characterize the physical properties of the 2DEG, both macroscopic and nanoscale electro-structural analysis of the near-surface region of the samples were performed. First, current-voltage (*I*-*V*) and capacitance-voltage (*C*-*V*) measurements of HEMT devices were performed in a Karl Süss probe station, equipped with a parameter analyzer. These macroscopic electrical measurements gave information on the current flowing in the 2DEG, allowing also to determine the threshold voltage and the sheet carrier density in the 2DEG. Then, TEM analysis was used to monitor the heterojunction microstructure and the crystalline defects. AFM and C-AFM were used to study the sample morphology as well as the local electrical behaviour of the modified surface region.

Finally, a preliminary investigation on the effect of a near-surface oxidation process was performed. For this aim, a rapid thermal oxidation (RTO) at 900°C for 10 min was carried out in a Jipelec JetFirst furnace. The nanoscale electro-structural properties of the oxidized region were characterized by means of TEM, AFM and C-AFM.

## Results and discussion

Figure 1c shows the *I*_DS_-*V*_GS _characteristics for different gate biases *V*_GS_, in the case of a reference untreated (as prepared) HEMT device (squares) and for a device subjected to a CHF_3 _plasma treatment (circles). For the untreated device a saturation current of 2.2 mA is reached at a gate bias *V*_GS _= 0, while at the same gate voltage (*V*_GS _= 0) the saturation current decreases to 0.15 mA in the CHF_3_-treated device. It is worth noting that a positive gate bias of +2 V must be applied to the HEMT subjected to CHF_3 _treatment to achieve a saturation current value of 2.4 mA, comparable with that in the untreated device at *V*_GS _= 0 V. Furthermore, the gate bias necessary to reduce *I*_DS _to a value of 10 nA changes from -2 to -0.5 V, from the untreated to the plasma-treated device. Finally, for a fixed gate bias of -2 V the leakage current decreases from 10 to 0.5 nA, after the plasma treatment.

**Figure 2:**
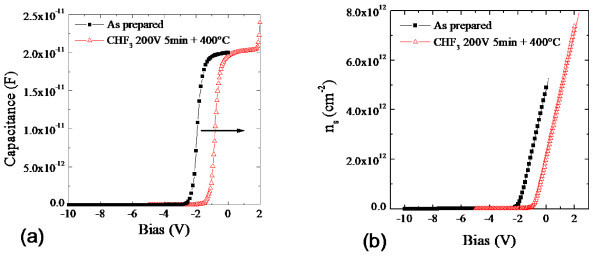
**Capacitance and sheet carrier density versus gate bias**. Capacitance versus gate bias (*C*-*V*_GS_) **(a) **and sheet carrier density versus gate bias (*n_s_*-*V*_GS_) **(b) **measured on the untreated (squares) and plasma treated (triangles) devices.

Figure 2a reports the *C*-*V*_GS _curves acquired in the same devices between the gate Schottky contact and the source electrode. A shift towards less negative values on the bias axis is visible for the *C*-*V*_GS _curve on the plasma-treated sample. The sheet carrier concentration *n*_s _can be also evaluated by integrating the *C*-*V*_GS _curves, as described in detail in reference [1]. The *n*_s_-*V*_GS _curves for the untreated and CHF_3_-treated samples are reported in Figure 2b. For a gate bias of 0 V, a decrease of *n*_s _from 5 × 10^12 ^cm^-2 ^in the as-prepared sample to 2 × 10^12 ^cm^-2 ^after the plasma treatment was found. For *V*_GS _= +2 V, *n*_s _reaches a value of 7 × 10^12 ^cm^-2^, for the plasma-treated sample. From the *n*_s_-*V*_GS _curves in Figure 2(b), it was also possible to extract a precise value of the threshold voltage. We found a *V*_th _= -1.92 V for the as prepared device and *V*_th _= -0.8 V for the processed device.

Moreover, from the values of source-gate current *I*_GS _(not showed) we observed a decrease of the current of leakage for the plasma-treated device under reverse bias. In particular, at *V*_GS _= -10 V the leakage current was reduced from 100 to 10 nA. The decrease in the reverse leakage current was also accompanied by a reduced forward current (i.e., from 10 to 4 mA at *V*_GS _= +3 V), most probably due to an increase of the series resistance. The decreasing of the leakage current can be due to several reasons: (1) an increase of the Schottky barrier height, (2) the depletion of the 2DEG channel, and (3) an increase in the resistivity in the upper shallow AlGaN layer due to lattice damage.

**Figure 3:**
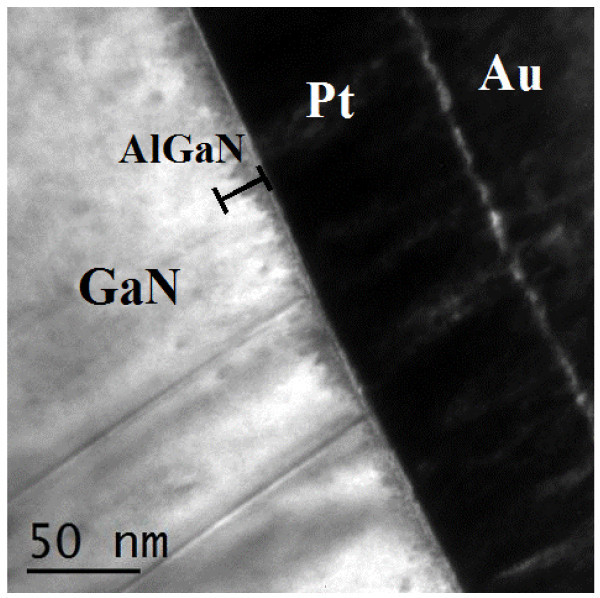
**TEM analysis of the heterojunction AlGaN/GaN after CHF**_**3 **_**plasma process**. A defect-rich region near the surface is visible.

Figure 3 shows cross-section TEM micrographs of our AlGaN/GaN heterostructure taken in the proximity of the gate of the HEMT device subjected to the plasma process. The dark contrast in the AlGaN region underneath the Pt gate contact can be associated to a considerable amount of crystalline imperfections (defects). This defect-rich interface region could be highly resistive and could affect the leakage current behaviour. Indeed also Chu et al. [10] suggested that the fluorine plasma can react with GaN (or AlGaN) to form non volatile F-containing compounds, leading to the creation of an insulating surface that blocks the leakage current.

**Figure 4:**
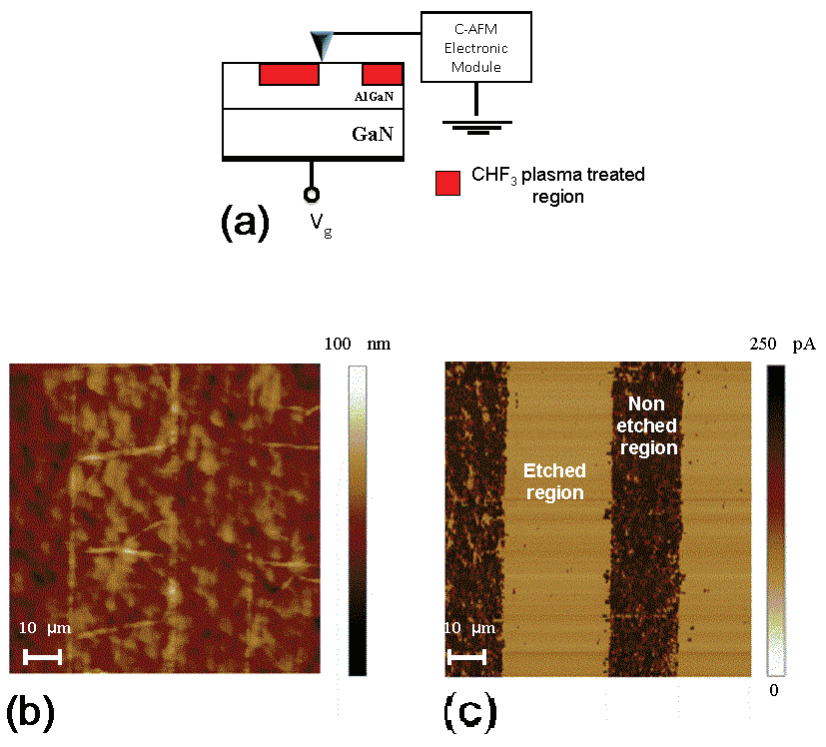
**C-AFM scans**. Schematic of the C-AFM measurement setup **(a) **used to measure conductivity changes in a sample locally treated with CHF_3 _plasma (on lithographically defined stripes) and annealed at 400°C. AFM morphology **(b) **and C-AFM transversal current map **(c) **of the sample.

In order to monitor the local electrical modification induced by the plasma treatment on the 2DEG, and corroborate the previous hypothesis, a nanoscale characterization approach was adopted. For this purpose C-AFM scans were performed on appropriate samples, in which the plasma treatments were performed in selected regions. In particular, resist stripes were defined on the sample surface by means of optical lithography, in order to selectively expose the sample surface to CHF_3 _process. The transversal current between the nanometric tip contact and the sample backside was measured by a high sensitivity current sensor in series with the tip, as illustrated in Figure 4a.

Figure 4b reports the AFM morphological image of the sample. As can be seen, no substantial difference can be observed between stripes processed with CH_3 _plasma and stripes without any treatment. On the other hand, a significant difference can seen by the transversal current map acquired by C-AFM and shown in Figure 4c. This picture clearly shows the electrical changes of the material due to the plasma treatment. The local current is significantly reduced (two orders of magnitude) on the stripes processed with plasma, with respect to the ones without plasma treatment. This behaviour is consistent with an increased local resistance in the plasma-etched regions, which in turn can be associated whether to a partial depletion of the 2DEG channel or more simply to an increase of the local resistance of the AlGaN barrier layer due to plasma-induced damage.

The experimental results found from the macroscopic I-V characteristic of the devices and the nanoscale electro-structural analysis of the near-surface region suggest that the observed electrical modifications are due both to the introduction of negative fluorine ions (as already reported in the literature) but also to the plasma-induced damage.

The near-surface modification induced by a RTO process was also monitored by combining TEM and scanning probe microscopy techniques.

**Figure 5:**
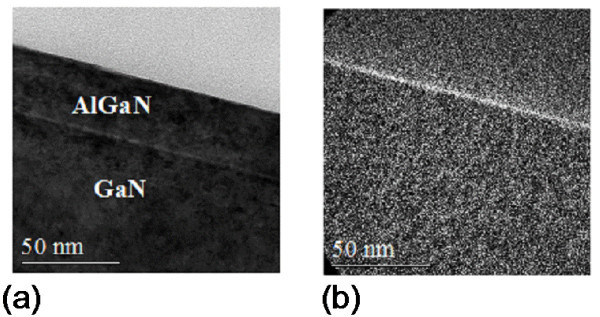
**TEM images of the oxidized sample**. Bright field TEM analysis **(a) **and EFTEM **(b) **for oxygen on a sample oxidized by RTA at 900°C for 10 min.

Figure 5 shows the TEM images of the oxidized sample. Combining the bright field image (a) with the oxygen map acquired by EFTEM (energy-filtered transmission electron microscopy) analysis (b) allowed to demonstrate the presence of a surface oxide layer of a thickness of about 2 nm grown after the process at 900°C. Previous experiments on long-term oxidation have shown the formation of a mixed oxide of Al_2_O_3 _- Ga_2_O_3 _with a high chemical stability with respect to wet etching [11].

**Figure 6:**
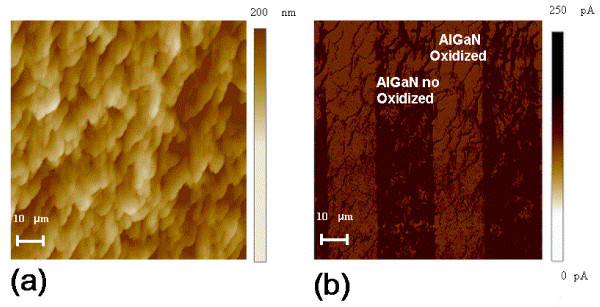
**Nanoscale electrical properties of the thin oxide formed by the RTO process monitored by C-AFM**. AFM image **(a) **and C-AFM image **(b) **of stripes on surface of AlGaN by RTA oxidized at 900°C for 10 min.

The nanoscale electrical properties of the thin oxide formed by the RTO process were monitored by C-AFM (reported in Figure 6).

Similarly to the case of the sample treated with plasma, also in the oxidized sample we prepared a sample for local electrical characterization. The sample consisted of regions (stripes) of locally oxidized material alternating with non-oxidized material. As can be seen, while the morphology of the oxidized regions remains practically unchanged with respect to the non-oxidized ones (Figure 6a), the current flow through the 2DEG was locally suppressed in the oxidized regions, which in turn exhibit a more resistive behaviour (Figure 6b).

Hence, this selective local oxidation process can be potentially useful to tailor the electrical properties of AlGaN barrier layers and/or as a novel approach for recessed-gate or insulated-gate technology for normally-off GaN HEMTs.

## Conclusion

In summary, a nanoscale approach was used to monitor the impact of near-surface processing on the electrical and structural properties of AlGaN/GaN heterostructures. The introduction of defects and/or negative charges by the CHF_3 _into the GaN (or AlGaN/GaN heterostructure) was deduced by TEM and C-AFM and can be indicated as the main cause of the depletion of the 2DEG and shift of the threshold voltage in HEMT devices.

A local increase of the resistivity was observed by a rapid thermal oxidation of the sample, which led to the formation of a very thin surface oxide. In this perspective, the nanoscale comprehension of the effects associated to the CHF_3 _plasma treatment and to oxidation processes can be useful to design and fabricate normally-off devices, with an insulated gate technology.

## Competing interests

The authors declare that they have no competing interests.

## Authors' contributions

GG carried out the electrical measurements, performed the electrical analysis and drafted the manuscript. FG carried out the AFM images and C-AFM current maps. AF contributed to the implementation of the electrical measurement. VR participated in the design of the study and its coordination.

FR planned the experiment, participated in its coordination, worked in data interpretation and drafted the manuscript. All authors read and approved the final manuscript.
